# Extracellular Hsp72, an endogenous DAMP, is released by virally infected airway epithelial cells and activates neutrophils via Toll-like receptor (TLR)-4

**DOI:** 10.1186/1465-9921-10-31

**Published:** 2009-04-30

**Authors:** Derek S Wheeler, Margaret A Chase, Albert P Senft, Sue E Poynter, Hector R Wong, Kristen Page

**Affiliations:** 1 Division of Critical Care Medicine, Cincinnati Children's Hospital Medical Center, Cincinnati Children's Research Foundation, Cincinnati, OH, USA; 2 Department of Pediatrics, University of Cincinnati College of Medicine, Cincinnati, OH, USA; 3 Infectious Diseases Program, Lovelace Respiratory Research Institute, Albuquerque, New Mexico, USA

## Abstract

**Background:**

Neutrophils play an important role in the pathophysiology of RSV, though RSV does not appear to directly activate neutrophils in the lower airways. Therefore locally produced cytokines or other molecules released by virally-infected airway epithelial cells are likely responsible for recruiting and activating neutrophils. Heat shock proteins (HSPs) are generally regarded as intracellular proteins acting as molecular chaperones; however, HSP72 can also be released from cells, and the implications of this release are not fully understood.

**Methods:**

Human bronchial epithelial cells (16HBE14o-) were infected with RSV and Hsp72 levels were measured by Western blot and ELISA. Tracheal aspirates were obtained from critically ill children infected with RSV and analyzed for Hsp72 levels by ELISA. Primary human neutrophils and differentiated HL-60 cells were cultured with Hsp72 and supernatants analyzed for cytokine production. In some cases, cells were pretreated with polymyxin B prior to treatment with Hsp72. IκBα was assessed by Western blot and EMSA's were performed to determine NF-κB activation. HL-60 cells were pretreated with neutralizing antibody against TLR4 prior to Hsp72 treatment. Neutrophils were harvested from the bone marrow of wild type or TLR4-deficient mice prior to treatment with Hsp72.

**Results:**

Infection of 16HBE14o- with RSV showed an induction of intracellular Hsp72 levels as well as extracellular release of Hsp72. Primary human neutrophils from normal donors and differentiated HL-60 cells treated with increasing concentrations of Hsp72 resulted in increased cytokine (IL-8 and TNFα) production. This effect was independent of the low levels of endotoxin in the Hsp72 preparation. Hsp72 mediated cytokine production via activation of NF-κB translocation and DNA binding. Using bone marrow-derived neutrophils from wild type and TLR4-mutant mice, we showed that Hsp72 directly activates neutrophil-derived cytokine production via the activation of TLR4.

**Conclusion:**

Collectively these data suggest that extracellular Hsp72 is released from virally infected airway epithelial cells resulting in the recruitment and activation of neutrophils.

## Introduction

The heat shock response is an ancient, highly conserved, endogenous cellular defense mechanism characterized by the rapid upregulation of a specific group of proteins called heat shock proteins [1]. While the heat shock response was first described nearly 50 years ago, it has only been relatively recently that these proteins have been linked closely with the host innate immune response [2]. For example, increased expression of the 72 kDa heat shock protein (Hsp72) appears to abrogate proinflammatory gene expression through inhibition of the transcription factor, NF-κB [3]. Heat shock proteins, such as Hsp72 and Hsp90 appear to be intimately involved in the recognition of so-called pathogen-associated molecular patterns (PAMPs), such as lipopolysaccharide (LPS). In this regard, Hsp72 and Hsp90 associate with Toll-like receptor (TLR)-4, CD14, and MD-2 within the lipid raft and appear to be crucial in the regulation of the assembly and subsequent function of the TLR-4 receptor complex [4-7]. Finally, the release of Hsp72 into the extracellular environment may serve to signal an impending danger signal to neighboring cells [8]. In this context, Hsp72 is now included in a growing list of so-called alarmins, a group of endogenous proteins that are released by necrotic cells or secreted via non-classical pathways in order to convey a danger signal to surrounding cells [9]. Endogenous alarmins, such as Hsp72, and exogenous PAMPs, such as LPS, both convey a similar message of danger that results in a patterned response. Together, alarmins and PAMPs comprise the so-called danger-associated molecular patterns (DAMPs) [10].

Collectively heat shock proteins are the most abundant intracellular proteins, representing up to 10% of the total protein content in the cell [11]. Hsp72 in particular is markedly induced in response to a diverse range of cellular insults [1,12], including increased temperature, oxidative stress, glucose deprivation, chemical exposure, ischemia-reperfusion injury, ultraviolet radiation, and infectious agents such as LPS. Therefore, Hsp72 or additional inducible stress proteins, by virtue of their relative abundance during times of stress are reliable markers of cell stress and plausible candidates for endogenous DAMPs.

We have recently shown that extracellular Hsp72 induces interleukin (IL)-8 expression in cultured human bronchial epithelial cells [13]. IL-8 is the principal neutrophil chemotactic cytokine ("chemokine") in humans and plays an important role in the pathophysiology of respiratory syncytial virus (RSV) lower respiratory tract infections (LRTI) in young children [14-17]. RSV is the leading cause of LRTI in children less than 1 year of age. More than 20% of all children will have RSV-associated wheezing during their first year of life, and 2–3% of these children will be hospitalized [18,19]. Approximately 120,000 children, most of whom are younger than 6 months of age, are hospitalized with RSV LRTI in the U.S. each year – close to 200 of these infants will die as a result of complications attributed to RSV [20]. Neutrophils are the predominant airway leukocytes found in children with RSV LRTI, though it appears that RSV does not directly activate neutrophils. Rather, proinflammatory cytokines and other molecules, possibly danger signals such as Hsp72, released by RSV-infected airway epithelial cells recruit and activate neutrophils in the lower airways [21]. Accordingly, we hypothesized that (i) RSV infection results in the release of extracellular Hsp72 from the airway epithelium and (ii) extracellular Hsp72 acts as a danger signal or alarmin, resulting in the subsequent recruitment and activation of neutrophils in the lung.

## Materials and methods

### Cell culture

SV40-transformed human bronchial epithelial cells (16HBE14o-) were grown as previously described [22]. HL-60 promyelocytic leukemia cells (ATCC, Manassas, VA) were cultured in RPMI 1640 medium supplemented with 10% fetal bovine serum, 50 mg/ml streptomycin, 2 U/mL penicillin, and 2 mM L-glutamine. For differentiation, HL-60 cells (1 × 10^6^/mL) were incubated in the presence of 1% DMSO for 3 days. 6 h prior to experimentation, cells were deprived of serum. In some cases, HL-60 cells were pretreated with polymyxin B (50 μg/ml for 1 h; Sigma, St. Louis, MO) or a TLR4 antibody (10 μg/ml for 1 h; eBiosciences, San Diego, CA) prior to treatment. Culture media from HL-60 cells treated with Hsp72 were analyzed for cytotoxicity using the Lactate Dehydrogenase (LDH) cytotoxicity BioAssay (US Biological) according to manufacturers' specifications.

### Generation of low endotoxin, human recombinant Hsp72

Recombinant Hsp72 was generated in our laboratory as recently described [23]. The Hsp72 preparation was purified using an endotoxin binding column (Pierce, Rockford, IL). Endotoxin levels (110 EU/mg Hsp72 protein or 11 ng/mg Hsp72 protein) were independently measured at Charles River Laboratories (Wilmington, MA).

### Preparation of RSV

HEp-2 cells were maintained in Eagle's minimal essential media (EMEM) supplemented with 10% fetal bovine serum, 2 mM L-glutamine, and 100 U/ml penicillin/streptomycin (10% EMEM). The A2 strain of RSV was plaque-purified three times under agarose. The third plaque was inoculated into a subconfluent HEp-2 cell monolayer. After adsorption for 1 h at room temperature, 10% EMEM was added and the infection was allowed to proceed for 3 d at 37°C until the entire monolayer showed cytopathic effects. The contents of the flask were resuspended and distributed in 1 ml aliquots, quick-frozen with alcohol/dry ice, and stored at -80°. Virus was derived from this master stock by infecting subconfluent HEp-2 monolayers at multiplicity of infection (MOI) of 0.1, and harvesting the monolayer when it appeared to be completely infected. The cells and media were sonicated (Ultrasonic homogenizer; Cole-Parmer Instrument Co., Chicago, IL) on ice with eight 1 s bursts using output of 50 and then the suspension was clarified by centrifugation at 1000 × *g *for 10 min. The supernatant was frozen and stored at -80°C and thawed rapidly at 37°C for use. Viral titers were determined by plaque assay.

### Viral infection

16HBE14o- cells were infected with RSV at an MOI of 1 to 10 for 24 hrs in media containing low IgG fetal bovine serum at 37°C in 5% CO_2_. Cell supernatant was collected and analyzed by ELISA for Hsp72 (Stressgen, Vancouver, BC) according to the manufacturer's protocol. Cells were lysed as previously described [13] for Western blot analysis of Hsp72. 16HBE14o- cells were infected with RSV at an MOI of 1 to 10 for 4 hrs and

### Western immunoblot

Whole cell lysates containing 30 μg of protein were resolved on a 8–16% Tris-glycine gradient gel and transferred to nitrocellulose. After incubation with primary antibody (Hsp72 from Stressgen, Vancouver, BC or IκBα from Santa Cruz Biotechnology, Santa Cruz, CA), signals were amplified and visualized using enhanced chemiluminescence.

### Collection of tracheal aspirates from infants with RSV LRTI

We prospectively enrolled all children less than 2 years of age who were admitted to our PICU with acute respiratory failure secondary to RSV bronchiolitis with approval by the Cincinnati Children's Hospital Medical Center Institutional Review Board (IRB) and following written informed consent from the parents' or legal guardians. Tracheal aspirates were obtained as previously described [24]. Briefly, tracheal aspirates were collected with routine suctioning by instilling a total of 3 mL of sterile 0.9% saline in 1 mL aliquots into the endotracheal tube. After installation, patients were hand-ventilated in order to disperse the saline, and the trachea was suctioned via a catheter placed slightly beyond the tip of the endotracheal tube. Multiple suction specimens were collected and pooled together and immediately refrigerated at 4°C. Samples were then centrifuged at 2000 rpm at 4°C for 5 minutes. The supernatant was collected and stored at -70°C until further analysis. Specimens were stored in aliquots in order to minimize the number of freeze-thaw cycles.

### Isolation of primary human neutrophil isolation

Following approval by the IRB and with informed consent, blood was collected using sterile technique from healthy volunteers for isolation of polymorphonuclear (PMN) leukocytes. Blood was collected into heparinized vacutainers and then subjected to Dextran T500 sedimentation, Ficoll-Histopaque density gradient centrifugation, and hypotonic erythrocyte lysis, as previously described [25]. PMNs were resuspended in serum deprived media for 4 h prior to exposure to treatment conditions.

### Isolation of primary mouse bone marrow-derive neutrophils

Femurs and tibias were removed from C3H/HeOuJ (wild type) and C3H/HeJ (spontaneous mutation in TLR4) mice were purchased from Jackson Laboratory and housed in a virus-free animal facility. Animal care was provided in accordance with National Institutes of Health guidelines. These studies were approved by the Cincinnati Children's Hospital Medical Center Institutional Animal Care and Use Committee. Bone marrow was isolated, rinsed and red blood cells are lysed. Resuspended cells are layered onto a three step Percoll gradient (52%, 64%, 72%) and centrifuged (1,000 × g for 30 min at RT). The bottom layer (64%–72%) contains the neutrophils and is collected, counted and plated.

### ELISA for quantification of cytokines

Following treatment, cell supernatants were collected and clarified (13,000 × g for 10 min at 4°C) prior to being analyzed for IL-8, KC or TNFα (R&D Systems, Minneapolis, MN) according to manufacturers' specifications.

### Quantitative real time PCR

16HBE14o- cells were infected with RSV at 5 and 10 MOI as described above for 4 h. Primary human neutrophils were treated with Hsp72 (30, 100 or 300 ng/ml) for 4 h. In both cases, RNA was extracted using a standard TRIzol method of phenol extraction. Total RNA is converted to cDNA by reverse transcription using the Superscript First Strand Synthesis System kit (Invitrogen, Carlsbad, CA). The Hsp72 (5'-3': AAG ATC TGC GTC TGC TTG GT and 3'-5': CGA CTT GAA CAA GAG CAT CA), TNFα (5'-3': AGG CCC CAG AGT TTT GTT CT and 3'-5': GGC AGC AGG TGG AAT TGT AT), IL-8 and SHDA primers which designed to span an intron, and the conditions of the real time run are as previously described [26]. Each target gene is normalized to a housekeeping or reference gene using the calculation (E ref)^Ct ref^/(E tar)^Ct tar ^; where E is the real time efficiency of the reference (ref) or target (tar) gene reaction and Ct is the threshold cycle of the reference (ref) or target (tar) gene.

### Nuclear protein extraction

Differentiated HL-60 cells were treated with Hsp72 (100 ng/ml) for 1 h. Cells were harvested and nuclear proteins were isolated as previously described [27]. All nuclear protein extraction procedures were performed on ice with ice-cold reagents. Protein concentrations of the resultant supernatants were determined using the Bradford assay. Nuclear proteins were stored at -70°C. The NF-κB probe (Santa Cruz, Santa Cruz, CA) was labeled with γ-^32^P adenosine triphosphate using T4 polynucleotide kinase (Invitrogen) and purified in Bio-Spin chromatography columns (BioRad). The gel was run using 10 μg of nuclear protein as previously described [27]. In some instances, antibodies against p65 (Rel A) or p50 (NF-κB1; Santa Cruz Biotechnology) were added (10 min at room temperature). Cold specific or nonspecific probes were added at 5× the concentration of the radiolabeled probe. Gels were transferred to Whatman 3 M paper, dried under a vacuum at 80°C for 1 hr, and exposed to photographic film at -70°C with an intensifying screen.

### Statistical analysis

When applicable, statistical significance was assessed by one-way analysis of variance (ANOVA). Differences identified by ANOVA were pinpointed by Student-Newman-Keuls' multiple range test.

## Results

### RSV infection induces Hsp72 expression in airway epithelial cells

**Figure 1:**
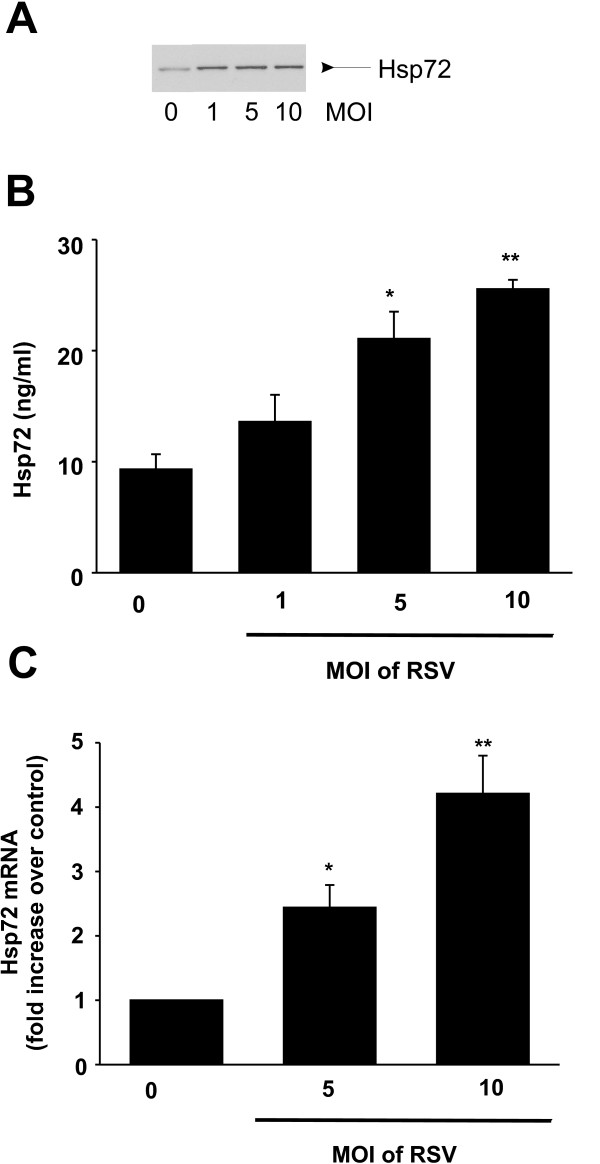
**RSV infection activates the stress response in human bronchial epithelial cells**. **A**. 16HBE14o- cells were infected with RSV at 1 – 10 MOI and intracellular proteins were harvested for Western immunoblot at 6 h. RSV infection resulted in a dose-dependent increase in intracellular Hsp72 expression. This experiment is representative of two separate experiments. **B**. 16HBE14o- cells were infected with RSV at 1 – 10 MOI for 18 h and supernatants were isolated. Hsp72 levels in the supernatant were assayed by ELISA. Data represent means ± SEM for 3 separate experiments and differences pinpointed by ANOVA. RSV infection resulted in a significant increase in extracellular Hsp72 release (* p = 0.002 and **p = 0.006 compared to uninfected). **C**. Quantitative real time PCR was expressed as Hsp72 normalized to SDHA and expressed as fold increase over control is shown (compared to uninfected *p = 0.044, **p = 0.003, n = 3).

Previous studies have suggested that RSV induces intracellular Hsp72 expression [28,29]. In order to confirm these results in our model, we infected 16HBE14o- cells with RSV at an MOI of 1 to 10 for 6 h and measured intracellular expression via Western immunoblot. RSV infection resulted in a dose-dependent increase in intracellular Hsp72 expression (Figure 1A). These results confirm that RSV infection activates the stress response *in vitro*. Next, we measured extracellular Hsp72 levels following RSV infection (MOI of 1 – 10). Again, RSV infection resulted in a dose-dependent release of extracellular Hsp72 (Figure 1B). We found that the preparation of RSV does in fact contain a negligible amount of Hsp72 (0.32 ng/μl). To confirm that RSV was indeed regulating intracellular Hsp72 synthesis, we performed quantitative real time PCR on 16HBE14o- cells infected with RSV at an MOI of 5 and 10. As shown in Figure 1C, infection with RSV increased mRNA levels for Hsp72, confirming that RSV infection is regulating endogenous Hsp72 production in human bronchial epithelial cells. Consistent with our previous data [30], neither monensin nor brefeldin A, both inhibitors of classic protein secretory pathways, had any appreciable inhibitory effect on extracellular Hsp72 release (*data not shown*). Together these data show that RSV infection increases both intracellular levels of Hsp72 and extracellular release of Hsp72. Furthermore, release of Hsp72 does not appear to be dependent on the classic protein secretory pathway in this model.

### Hsp72 is increased in tracheal aspirates from RSV-infected children

**Figure 2:**
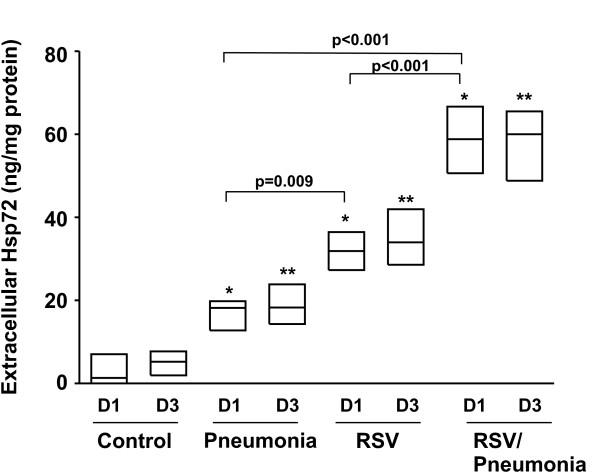
**Extracellular Hsp72 in tracheal aspirates of critically ill children**. Tracheal aspirates from children with pneumonia, RSV, RSV with pneumonia, or controls without bacterial or viral infection on Day 1 (D1) or Day 3 (D3) of hospitalization. Tracheal aspirates were clarified and analyzed for Hsp72 levels by ELISA. In each case, bars represent each sample from 5–12 patients and the line represents the mean and differences pinpointed by ANOVA (compared to D1 control *p < 0.001; compared to D3 control **p < 0.001). Statistics are also shown to compare the disease groups.

To determine the clinical relevance of our *in vitro *cell culture studies, we obtained tracheal aspirate samples from 7 healthy controls who were tracheally intubated and mechanically ventilated following airway reconstructive surgery, 8 critically ill children with acute respiratory failure secondary to documented bacterial pneumonia (defined as a positive respiratory culture or blood culture with lobar infiltrates on chest radiograph), 12 critically ill children with acute respiratory failure secondary to RSV LRTI, and 5 critically ill children with acute respiratory failure secondary to RSV LRTI complicated by bacterial pneumonia (Table 1). The healthy controls were significantly older and had lower Pediatric Risk of Mortality-II (PRISM-II) [31] scores compared to the critically ill children, though there were no other differences between the critically ill children with RSV LRTI, bacterial pneumonia, or RSV LRTI complicated by bacterial pneumonia and healthy controls. As shown in Figure 2, critically ill children with acute respiratory failure secondary to RSV LRTI had a significant elevation in extracellular Hsp72 compared to otherwise healthy children without RSV. In addition, at day 1 children with RSV LTRI complicated by bacterial pneumonia had statistically higher levels of extracellular Hsp72 than children with only RSV LTRI or only bacterial pneumonia. Collectively, these data suggest that RSV infection activates the stress response, increasing both the intracellular expression and extracellular release of Hsp72 and further provide clinical relevance to our *in vitro *data.

**Table 1:** Demographic characteristics of 25 critically ill children with acute respiratory failure and 7 healthy controls with post-operative respiratory failure.

**Demographic**	**Control (n = 7)**	**Bacterial pneumonia (n = 8)**	**RSV LRTI Alone (n = 12)**	**RSV LRTI + Pneumonia (n = 5)**
Age, median (IQR)	16 mos (13–18)*	4 mos (1–6)	2 mos (1–4)	2 mos (1–2)

Gender, M:F	4:3	5:3	4:8	2:3

PRISM-II score, mean ± SEM	0.43 ± 1.0*	5.3 ± 1.2	3.6 ± 1.0	7.6 ± 1.2

Ventilator Days, median (IQR)	7 (5–7)	5 (4–8)	7 (6–8)	8 (7–10)

Tracheal aspirate samples were isolated from healthy controls, critically ill children with acute respiratory failure secondary to either bacterial pneumonia, RSV lower respiratory tract infection (LRTI) or RSV LRTI complicated by bacterial pneumonia. Age in months, gender, Pediatric Risk of Mortality (PRISM)-II scores and median ventilator days are shown. * p < 0.05 compared to critically ill children with bacterial pneumonia, RSV LRTI, or RSV LRTI + bacterial pneumonia.

### Extracellular Hsp72 increases IL-8 and TNFα production in neutrophils

**Figure 3:**
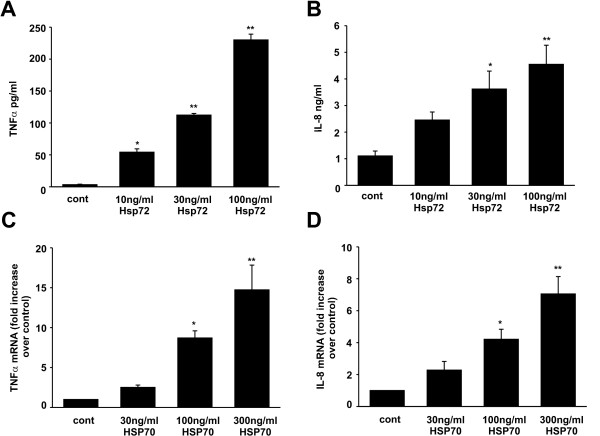
**Exogenous Hsp72 increased cytokine production in primary human neutrophils**. Primary human neutrophils were harvested, isolated, and treated with increasing concentrations of Hsp72 for 4 (quantitative real time PCR) or 18 (ELISA) hours. Data represent means ± SEM for 3–4 experiments and differences pinpointed by ANOVA. **A**. TNFα ELISA (compared to control, *p = 0.002, **p < 0.001). **B**. IL-8 ELISA (compared to control *p = 0.041, **p = 0.004). Quantitative real time PCR was expressed as IL-8 or TNFα normalized to SDHA and expressed as fold increase over control is shown. **C**. TNFα mRNA levels (compared to control *p = 0.02, **p = 0.001). **D**. IL-8 mRNA levels (compared to control *p = 0.02, **p < 0.001).

**Figure 4:**
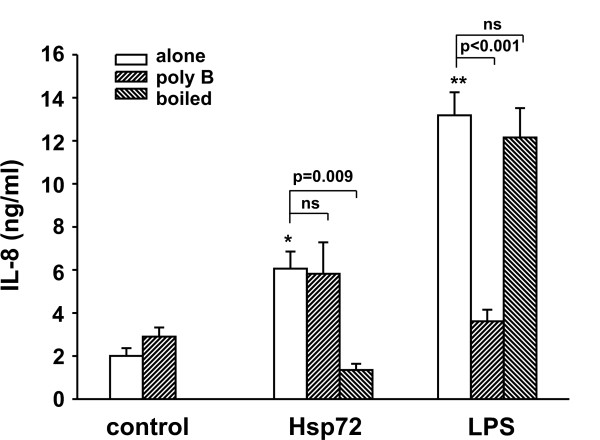
**Exogenous Hsp72 activates IL-8 production in HL-60 cells in an endotoxin-independent manner**. Differentiated HL-60 cells were pretreated with polymyxin B (50 μg/ml) for 1 h prior to treatment with Hsp72 (100 ng/ml) or LPS (100 ng/ml). In some cases, cells were treated with Hsp72 or LPS which had been boiled for 1 hr. Supernatants were harvested, clarified and run on IL-8 ELISA. Data represent means ± SEM for 4 experiments and differences pinpointed by ANOVA (compared to untreated control *p = 0.002, **p < 0.001).

We have previously shown that extracellular Hsp72 induces IL-8 gene expression in 16HBE14o- cells [13]. Neutrophils play an important role in the pathophysiology of RSV LRTI, though RSV does not appear to directly activate neutrophils in the lower airways [21]. Locally produced cytokines or other molecules released by virally infected airway epithelial cells are likely responsible for recruiting and activating neutrophils. We therefore treated primary human neutrophils from normal donors with increasing concentrations of recombinant Hsp72. Treatment with Hsp72 increased TNFα and IL-8 protein levels in a dose-dependent manner (Figure 3A and 3B). Real time PCR confirmed that Hsp72-induced significantly increased TNFα and IL-8 mRNA levels (Figure 3C and 3D). We also tested the effects of Hsp72 on DMSO-differentiated HL-60 cells and found a similar level of IL-8 activation. Hsp72 treatment did not alter HL-60 cell cytotoxicity as determined by lactose dehydrogenase (LDH) release into the culture media (10.4 ± 1.2% cytotoxicity in the control compared to 11.2 ± 0.8% cytotoxicity in the Hsp72-treated HL-60 cells; n = 4, p = 0.63). As an important control to rule out confounding effects from any potential endotoxin contamination, we pretreated cells with polymyxin B to bind endotoxin prior to Hsp72 and LPS treatment, and we boiled Hsp72 and LPS prior to addition to cells. Polymyxin B treatment did not alter IL-8 expression in Hsp72 treated cells but significantly decreased LPS-induced IL-8 expression (Figure 4), suggesting that extracellular Hsp72 itself, and not endotoxin contamination of the recombinant protein, induces IL-8 gene expression in human neutrophils. In addition, boiled Hsp72 was unable to activate IL-8 production, while boiled LPS still induced IL-8 cytokine expression, further showing that Hsp72 and not contaminating endotoxin was responsible for the effect on cytokine production. Since we were obtaining similar cytokine regulation using HL-60 cells compared to primary human neutrophils, we performed the remaining mechanistic studies using HL-60 cells.

### Neutrophil activation by extracellular Hsp72 is dependant upon the NF-κB pathway

**Figure 5:**
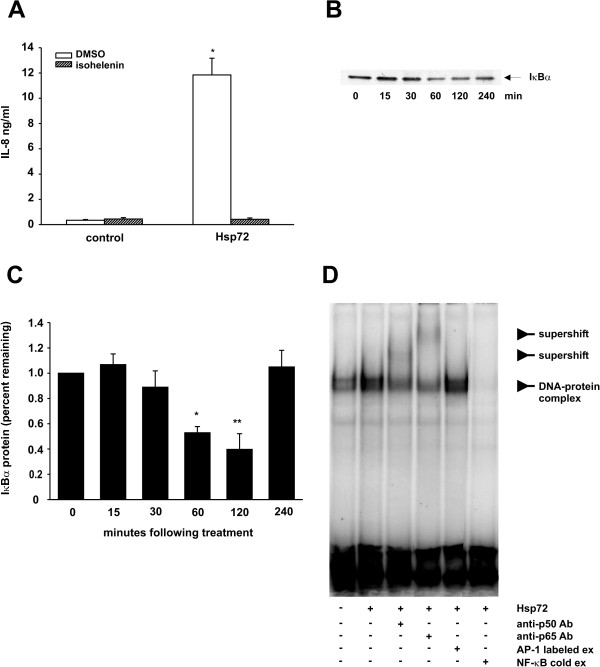
**Exogenous Hsp72 activates NF-κB in HL-60 cells**. **A**. Differentiated HL-60 cells were pretreated with isohelenin (30 μM) or DMSO for 1 h prior to treatment with Hsp72 (100 ng/ml). Supernatants were harvested, clarified and run on IL-8 ELISA. Data represent means ± SEM for 3 experiments and differences pinpointed by ANOVA (compared to control *p < 0.001). **B**. HL-60 cells were treated with Hsp72 (100 ng/ml) for times as indicated (0–240 minutes). Cell lysate was harvested, run on a Western blot and probed for IκBα. Shown is a representative Western blot. This experiment was performed 4 times. **C**. Quantification of the IκBα western blots. Data are expressed as percentage of IκBα remaining compared to untreated sample for each experiment (mean ± SEM for 4 experiments *p = 0.014, **p = 0.005). **D**. Nuclear extracts from cells treated with or without Hsp72 (100 ng/ml) were incubated with an oligonucleotide NF-κB consensus sequence and EMSA was performed. Antibodies against p65RelA or p50 NF-κB were added to selected samples. Cold NF-κB probe (5× excess) or cold AP-1 probe (5× excess) was added to a sample to show specificity. These data are representative of two separate experiments.

We initially investigated the role of NF-κB in Hsp72-induced cytokine production using a chemical inhibitor of the NF-κB pathway. Pretreatment of HL-60 cells with the NF-κB inhibitor isohelenin completely abolished Hsp-72-induced IL-8 expression (Figure 5A). Next, we asked whether Hsp72 regulated IκBα degradation. Treatment of HL-60 cells with Hsp72 lead to rapid degradation of IκBα followed by resynthesis of IκBα by 4 hours (Figure 5B and 5C). The kinetics of IκBα are consistent with the fact that IκBα is rapidly degraded to allow for NF-κB translocation to the nucleus, and then rapidly resynthesized to terminate the NF-κB signal. Next, we treated cells with Hsp72 for 1 hr and nuclear extracts were harvested for EMSA. Hsp72 increased the binding of nuclear proteins to an oligonucleotide encoding the consensus sequence NF-κB binding site. Co-incubation of nuclear extracts with antibodies against p65 RelA and p50 NF-κB1 each induced a supershift of the DNA binding complex, demonstrating the presence of these NF-κB family transcription factors (Figure 5D). Together these data implicate Hsp72 in the induction of NF-κB activation, translocation and DNA binding, which ultimately regulates cytokine production.

### Hsp72 regulates cytokine production via TLR4

**Figure 6:**
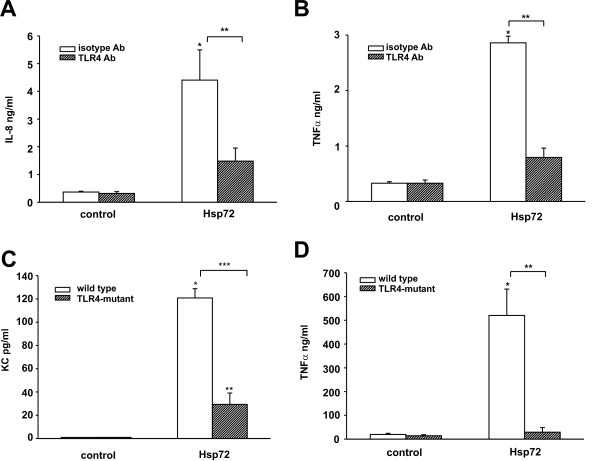
**TLR4 is required for Hsp72-induced regulation of IL-8 production from HL-60 cells**. Differentiated HL-60 cells were pretreated with a neutralizing antibody against TLR4 (10 μg/ml) or an isotype control antibody for 1 h prior to Hsp72 treatment (100 ng/ml). 18 h later, cell media was harvested and analyzed by ELISA for IL-8 and TNFα production. Data are expressed as mean ± SEM for 5 experiments and differences pinpointed by ANOVA. A. IL-8 ELISA. Compared to isotype antibody control *p = 0.004, compared to TLR4 antibody **p = 0.014. B. TNFα ELISA. Compared to isotype antibody control *p < 0.001, compared to TLR4 antibody **p < 0.001. Bone marrow-derived neutrophils were isolated from wild type (C3H/HeOuJ) or mice with a spontaneous mutation in TLR4 (C3H/HeJ). Primary neutrophils were treated *ex vivo *with Hsp72 (100 ng/ml) and supernatant was removed 18 h later for analysis by KC (the functional IL-8 in mouse) and TNFα ELISA. Data are expressed as mean ± SEM for 3 separate experiments and differences pinpointed by ANOVA. C. KC ELISA. Compared to control *p = 0.002, compared to Hsp72 **p < 0.001. D. TNFα ELISA. Compared to control *p < 0.001, compared to Hsp72 **p < 0.001.

Neutrophils express TLR4 as determined by RT-PCR and flow cytometry (data not shown). To assess the role of TLR4 in Hsp72-mediated cytokine production, we pretreated differentiated HL-60 cells with a neutralizing antibody against TLR4 for 1 hr prior to treatment with Hsp72. Hsp72-mediated IL-8 and TNFα expression was inhibited by the TLR4 neutralizing antibody, but not the isotype control antibody (Figure 6A and 6B). To further investigate the importance of TLR4 in mediating Hsp72-induced cytokine production in neutrophils, we harvested neutrophils from the bone marrow of wild type mice (C3H/HeOuJ) or mice with a spontaneous mutation of TLR4 which blocks TLR4 activation (C3H/HeJ). Neutrophils from wild-type mice had increased KC (the function homolog of IL-8 in mice) and TNFα expression when treated *ex vivo *with Hsp72 (Figure 6C and 6D). In contrast, the neutrophils from TLR4-mutant mice did not increase TNFα expression following Hsp72 treatment, suggesting the important role of TLR4 in Hsp72-induced cytokine expression.

## Discussion

Herein we show that RSV infection activates the local stress response in airway epithelial cells, as determined by increased intracellular production of the 72 kDa heat shock protein, Hsp72. RSV infection further results in the release of extracellular Hsp72, an important endogenous danger signal or alarmin [2]. These *in vitro *data are further corroborated by the clinical data, in which we demonstrate increased extracellular Hsp72 levels in the pulmonary edema fluid of critically ill children with acute respiratory failure secondary to RSV bronchiolitis. The tracheal aspirates in the healthy controls were obtained from children who were electively tracheally intubated and mechanically ventilated following airway reconstruction therapy, so these children were older than and not as sick as the critically ill children with acute respiratory failure secondary to bacterial pneumonia or RSV LRTI. While children appear to have a more robust increase in Hsp72 expression compared to adults [2,32], the differential Hsp72 expression in neonates versus young infants is not currently known. In separate experiments, we further show that extracellular Hsp72 increases IL-8 gene expression in neutrophils. Similar to what we have previously shown in human airway epithelial cells and mouse tracheal epithelial cells [13], Hsp72-induced IL-8 gene expression in neutrophils is dependent upon activation of both TLR-4 and NF-κB. Collectively, these results suggest that the release of Hsp72 by virally infected cells may serve as an important endogenous danger signal to activate the host innate immune response.

The innate immune response evolved to recognize pathogen associated molecular patterns (PAMP) which are common to many classes of pathogens. PAMPs are recognized by pathogen-recognition receptors, which include the toll like receptors (TLR). TLRs recognize specific components conserved among microorganisms. For example, peptidoglycan and lipotechoic acid from Gram positive bacteria signal through TLR2 while Gram negative bacterial lipopolysaccharide signals through TLR4. Moreover, TLR4 appears to be crucial to the initiation of the innate immune response to RSV infection. For example, TLR4-deficient mice are highly susceptible to RSV infection *in vivo *[33,34]. Consistent with these data, TLR4 gene polymorphisms have been associated with an increased risk of severe RSV LRTI in young infants and children [35-37]. Finally, RSV infection upregulates TLR4 expression in the airway epithelium *in vitro *[38] and *in vivo *[39]. Collectively, these data suggest that TLR4 plays an integral role in the pathobiology of RSV LRTI.

According to the danger model proposed by Matzinger [8], PAMPs such as LPS initiate the host immune response only if there is evidence of cellular injury, as indicated by the presence of so-called danger signals or alarmins. Several potential endogenous danger signals have been described, including uric acid [40], HMGB-1 [41], ATP [42], and heat shock proteins [2,23,30,41]. Heat shock proteins, such as Hsp72 would appear to be particularly well suited to act as endogenous danger signals or alarmins. Heat shock proteins are ancient, highly conserved molecules that have been identified in virtually every cell type and every organism, both prokaryotic and eukaryotic, that have been examined to date. In comparison, the exogenous danger signal, LPS appeared relatively late on the evolutionary time-scale and is much less ubiquitous, being unique only to gram-negative bacteria. Given the stark similarities between extracellular Hsp72-mediated and LPS-mediated signal transduction pathways, it is tempting to speculate that the programmed response to the exogenous danger signal, LPS, is modeled on the more primitive programmed response to the exogenous danger signal, extracellular Hsp72 [11]. Hsp72 is highly stress inducible and appears to be released from stressed or damaged cells, either through an as yet undefined secretory mechanism, non-specific release during cell necrosis, or a combination of both [30]. Once in the extracellular milieu, Hsp72 can act in either an autocrine or paracrine manner to increase pro-inflammatory gene expression via TLR4 [13,30].

The danger model is particularly relevant to the pathobiology of RSV LRTI. Neutrophils play an important role in RSV-mediated inflammation and injury [43,44]. For example, neutrophils comprise over 90% of inflammatory cells recovered from the upper airways and over 75% of inflammatory cells recovered from the lower airways in children with RSV LRTI [45]. RSV infection of the respiratory epithelium increases neutrophil adhesion and activation, which in turn further augments damage and detachment of the respiratory epithelial cells infected with the virus [46]. However, RSV does not appear to directly activate neutrophils in the lower airways [21]. Rather, locally produced cytokines or other molecules – perhaps endogenous danger signals, such as Hsp72 – released by virally infected airway epithelial cells are likely responsible for recruiting and activating neutrophils.

Based on these data, we propose that RSV infection increases activation of stress response pathways in the airway epithelium, resulting in the increased production and subsequent release of Hsp72. Extracellular Hsp72, in turn, increases IL-8 gene expression in surrounding airway epithelial cells in a TLR4- and NF-κB-dependent manner [13]. We cannot rule out the involvement of TLR2 in this process. We have preliminary data suggesting that cytokine expression and neutrophilia in the airways of mice following inhalation of Hsp72 were partially reduced in TLR2 knockout mice and that blocking TLR2 with an antibody prior to treatment with Hsp72 resulted in attenuated cytokine production from HL-60 cells (K. Page, unpublished observation). We are currently investigating the interactions of Hsp72 and TLRs, and we anticipate that Hsp72 will also activate TLR2. We have no evidence of Hsp72 activating any other TLR. We have previously shown that inhaled Hsp72 induced significant neutrophilia [13], however in this study we did not investigate whether the IL-8 or the Hsp72 released from airway epithelium was responsible for the recruitment of neutrophils into the airways. We did show in this study, however, that Hsp72 can directly affect neutrophil-derived cytokine production in a TLR4- and NF-κB-dependent manner. To our knowledge, the direct effects of extracellular Hsp72 on neutrophil chemotaxis and activation have not been previously studied [47], though *in vitro *studies have suggested a potential chemotactic role for membrane-bound Hsp72 for NK cells [48]. It is therefore plausible that extracellular Hsp72 derived from virally-infected epithelial cells could play an important role in both neutrophil recruitment and activation. Future studies will be necessary to further address these questions and further define the role of extracellular Hsp72 in the pathobiology of RSV LRTI.

## Conclusion

Collectively, the results of the current study suggest that extracellular Hsp72 is released from virally infected airway epithelial cells resulting in the recruitment and activation of neutrophils. These data provide additional support for our hypothesis that extracellular Hsp72 serves as an important endogenous danger signal. Further studies on the role of extracellular Hsp72 in the pathobiology of RSV LRTI are warranted.

## List of Abbreviations

DAMP: danger-associated molecular pattern; Hsp: heat shock protein; IL: interleukin; LPS: lipopolysaccharide; LTRI: lower respiratory tract infection; PAMP: pathogen-associated molecular pattern; RSV: respiratory syncytial virus; TLR: toll like receptor; TNF: tumor necrosis factor.

## Competing interests

The authors declare that they have no competing interests.

## Authors' contributions

DSW helped conceive the study and drafted the manuscript. MAC performed the neutrophil experiments. APS made the RSV and performed the RSV infection. SEP collected and analyzed the human samples. HRW consulted on the manuscript. KP conceived and coordinated the study, and helped draft and revise the manuscript. All authors read and approved of the final manuscript.
